# The Influence of Organizational Factors on Road Transport Safety

**DOI:** 10.3390/ijerph15091938

**Published:** 2018-09-06

**Authors:** Nuria Gamero, Inmaculada Silla, Rubén Sainz-González, Beatriz Sora

**Affiliations:** 1 Department of Social Psychology, University of Seville, 41004 Seville, Spain; ngamero@us.es; 2 CIEMAT-CISOT (Sociotechnical Research Centre), 08007 Barcelona, Spain; 3 Department of Economy, University of Cantabria, 39005 Santander, Spain; sainzru@unican.es; 4 Faculty of Psychology and Education Sciences, Open University of Catalonia, 08018 Barcelona, Spain; bsora@uoc.edu

**Keywords:** road transport safety, sociotechnical approach, organizational learning, training

## Abstract

Road transport safety is a major concern across Europe due to the human and socio-economic costs associated with work-related traffic accidents. Traditional approaches have adopted regulatory and technical measures to prevent road accidents leaving aside the organizational factors that might contribute to road transport safety. However, contemporary sociotechnical systems theory acknowledges the need to take into account organizational factors. This study adopts a sociotechnical approach and it examines the relationship between a number of organizational factors (organizational learning and training) and road traffic accidents in the organizations under study. Our sample was composed of 107 road transportation organizations from Spain. Binary logistic regression analyses were carried out to test our hypotheses. Organizational size and type of transport (goods or passengers) were included in the model as control variables. Results showed that in those organizations where organizational learning was supported, the occurrence of traffic accidents was less likely. Unexpectedly, the relationship between training and the occurrence of traffic accidents was not significant. Thus, findings partially supported the formulated hypothesis. Future research should shed light on the relationship between training and traffic accidents taking into account potential intervening variables.

## 1. Introduction

According to the European Road Safety Observatory [1], traffic accidents are a leading cause of work-related death and long-term injury. In particular, approximately 40–60% of work-related accidents resulting in death are road crashes. These accidents cause substantial socio-economic costs for countries, employers, and employees. Thus, research on how to prevent work-related traffic accidents is worthwhile and requires attention from organizations not only governments. In particular, this study focuses on how to prevent traffic accidents among professional drivers because it is a highly hazardous occupation [1].

Member States have developed safety standards and regulations that address road safety across Europe [2,3]. However, it is necessary to adopt a more comprehensive approach that goes beyond regulatory and technological approaches [4]. Along these lines, this study adopts a sociotechnical approach which acknowledges the role of organizational policies and practices to improve road safety.

In particular, this study addresses the relationship between the implementation of organizational practices that support organizational learning and training, and the occurrence of traffic accidents. This study was carried out in organizations dedicated to goods and passengers transport settled in Spain.

A sociotechnical approach to road safety. Contemporary sociotechnical systems theory has become a useful framework to better understand the complexity of transport safety and, subsequently, to develop and improve road safety policies and practices [4]. According to this paradigm, safety is an emergent property of the system [5]; thus, it can only be understood taking into account the system as a whole not their individual components in isolation. It implies that all the relevant actors must be taken into account. Moreover, reductionist approaches that attempt to understand traffic accidents and their causes based merely on local factors at the sharp end of system operation such as drivers’ behavior [6,7] are no longer valid [8]. The social environment in a broad sense (e.g., governmental policies and regulations, market economic and production pressures…) and organizational factors (e.g., organizational processes, human resources management policies…) must be taken into account to better understand safety in complex systems [9].

Despite many authors [4,10,11,12] have claimed the need to overcome reductionist approaches, little is known about organizational factors that explain traffic accidents among professional drivers. This study attempts to respond to this research gap by means of testing the relationship between several organizational processes (organizational learning and training) and traffic accident occurrence in the organizations under study.

Learning is defined as a process that enables the organization to create and manage knowledge outcomes [13] which allows continuous improvement [14]. Thus, it is expected to prevent incidents recurrences, but also to make an organization inherently safer [15]. Along these lines, many authors [16,17,18] have emphasized the relevance of organizational learning to promote safety and prevent accidents across different sectors such as the construction [19], the chemical industry [20], or the healthcare sector [21,22,23,24]. Despite this, to our knowledge, the relationship between organizational learning and traffic accidents remains unexplored.

With respect to training, it refers to the set of activities aimed at the acquisition of knowledge, skills, and attitudes which have an immediate or a short-term application (e.g., safe driving) [25]. Drivers’ training involves the improvement of driving skills and practices. It also helps employees to identify traffic hazards and enhances their road safety awareness. This study conceptualizes training as the continuous acquisition of knowledge and skills aimed at improving employees’ performance.

Previous research on the influence of training on traffic accidents is scarce [26] and its effectiveness has been questioned [27,28,29]. Additionally, it is noteworthy that previous research has mainly focused on the influence of target-specific training interventions [30] rather than periodic training understood as a continuous activity promoted by the organization. Thus, research on the influence of continuous training on road safety is needed. Along these lines, the European Parliament [31] has recently emphasized the need to improve periodic in-service training of professional drivers as a mean to promote road safety standards.

Moreover, despite professional driving (e.g., truck, bus or taxi drivers) is a highly hazardous occupation [1], to our knowledge, previous research has not examined if training reduces professional drivers’ likelihood of having a traffic accident [26]. Some studies have focused on other target groups that are also considered to be at risk of traffic accident. For instance, licensed drivers with poor driving records (prior crashes and/or offences) [26] or novice young drivers [29]. Nonetheless, those studies have focused on the influence of target-specific training programs that attempt to address their target groups’ needs. In particular, novice young workers are thought to be inexperienced and more likely to engage in risky and sensation seeking behaviors than older drivers; thus, they might benefit from “insight” and “awareness” based training which improves self-assessment of one’s own driving abilities [29]. Along these lines, empirical evidence has found that this kind of training marginally reduces traffic accidents among novice young drivers [29]. However, these findings cannot be extrapolated to professional drivers who deserve particular attention that take into account their work environment as postulated by sociotechnical systems theory. This study extends previous research by means of testing the influence of training, understood as a continuous activity promoted by the organization rather than a target-specific training intervention, on the occurrence of traffic accidents in a sample of professional drivers dedicated to goods and passengers transport.

Based on the aforementioned theoretical and empirical evidence, the following hypotheses were formulated:

**Hypothesis** **1.** Organizational learning will be negatively associated with the occurrence of traffic accidents in road transport organizations.

**Hypothesis** **2.** Training will be negatively related to the occurrence of traffic accidents in road transport organizations.

## 2. Materials and Methods 

### 2.1. Procedure and Sample

Our sample was composed of organizations from the logistics and road transport industry in Spain. These organizations were randomly selected from an extensive list of companies provided by the National Chamber of Commerce. The human resources managers of several organizations were then contacted and asked to participate in the study. Those who agreed were contacted telephonically and confidentiality was assured. Most questionnaires were completed during working hours.

The final sample was composed of 107 organizations (See Table 1). The response rate was 65.2%. Of these, 60.7% of the organizations were dedicated to goods transport and the remaining 39.3% to passenger transport. Most organizations had less than 50 employees (82.2%), 15% were medium size (between 50 and 250 employees), and only 2.8% had more than 250 employees. 

Most organizations had a similar structure and they performed similar functions. In most cases, road transport organizations were composed of a managing director, a Human Resources (HR) manager (sometimes, the managing director and the HR manager were the same person), a small number of administrative staff who performed administrative and financial tasks, several drivers and a small number of mechanics. This structure may vary depending on the organizational size. Regarding their staff background characteristics, 53.5% of the employees were between 25 and 40 years old. With regard to employees’ nationality, 35% were European citizens.

### 2.2. Measures

This study was part of a bigger project that addressed the main characteristics of the road transport organizations, services, structure, vehicle fleet, staff’s background characteristics, human resources policies, and occupational safety. As follows, we describe only those variables included in the current study.

*Traffic accident occurrence.* Human resources managers were asked about the occurrence of traffic accidents in their organizations during the previous year: “*Did any traffic accident take place in your organization last year?*” Respondents answered using a dichotomous scale (1—*Yes*; 0—*No*): 11.2% of organizations reported that have had a traffic accident during the previous year.

*Organizational Learning.* It was measured through a reduced version of 4 items of the Marsick and Watkins’s scale [14]. Sample items were “Time is devoted to finding ways of improvement” and “Employees are encouraged to view problems as learning opportunities”. Respondents answered using a five-point scale from 1 (*completely disagree*) to 5 (*completely agree*). Mean and standard deviation for this variable were 3.11 and 1.05 respectively. Cronbach’s α coefficient for this scale was 0.91.

*Training*. This variable was measured using a 3-item scale [32]. We used a reduced version of Delery and Doty’s scale. The items were preceded by the following question: “Could you tell me whether in your organization…?” A sample item was: “Employees attend a training course every one or two years” Respondents answered using a five-point scale from 1 (*not at all*) to 5 (*very much*). Mean and standard deviation for this variable were 2.97 and 1.51 respectively. Cronbach’s α coefficient was 0.94.

*Control variables*. We included two control variables (organizational size and type of transport) to control for potential confounding effects. We measured these control variables by asking human resources managers: *“How many people are there in your organization?”* (Organizational Size) and *“Does your organization transport goods or passengers?”* (Type of transport). The response scale of the organizational size variable ranged from 1 (“1–5 employees”) to 8 (“More than 500 employees”). In order to guarantee the parsimony of the model, organizations were categorized in three groups: small size (1–50 employees), medium (51–250 employees) and large organizations (More than 250 employees). The type of transport variable was operationalized as a dummy variable (1—goods; 2—passengers).

### 2.3. Data Analysis

As a prior step to testing our hypothesis, we calculated bivariate correlations. Moreover, hypotheses 1 and 2 were tested by using binary logistic regression analyses. Binary logistic regression is an extension of multiple regression analyses with the exception that the outcome variable is binomial [33]. All statistical data analyses were performed using SPSS version 24.0 [34].

Organizational size and type of transport were introduced in the regression model as control variables. Organizational size was controlled for several reasons. Our dependent variable measured traffic accident occurrence (“*Did any traffic accident take place in your organization last year?*” *Yes/No*) because number of accidents per organization adjusted for number of employees (yearly accident rate) was not available. Thus, we controlled for organizational size as a countermeasure to prevent potential confounding effects. With respect to type of transport, goods and passengers transport organizations face different regulations and market pressures. Subsequently, these organizations might adopt different organizational policies and practices (e.g., training).

## 3. Results

Table 2 shows correlations among the variables included in this study.

Correlations showed a negative and significant relationship between organizational learning and the occurrence of traffic accidents (*r* = −0.21, *p* < 0.05). Moreover, organizational learning displayed a positive and significant association with training (*r* = 0.57, *p* < 0.01).

Logistic regression model were conducted to predict traffic accident occurrence in road transport organizations. Hosmer-Lemeshow goodness of fit test for logistic regression displayed a non-significant chi-square value (*χ*^2^ = 5.903, *df* = 8, *p* = 0.658) which indicates that the model adequately fit the data. A summary of results from the binary logistic regression model are presented in Table 3. As postulated in hypothesis 1, organizational learning presents a negative and statistically significant relationship with traffic accident occurrence. In those road transport organizations where organizational learning is promoted, the occurrence of traffic accidents is less likely. Thus, findings support Hypothesis 1. Hypothesis 2 postulated that training would be negatively related to traffic accidents. However, the relationship between training and traffic accidents turned to be non-significant. Thus, Hypothesis 2 was not supported.

## 4. Discussion

The present study examined the relationship between organizational factors –organizational learning and training- and traffic accident occurrence in road transport organizations settled in Spain. As hypothesized (Hypothesis 1) findings supported the relationship between organizational learning and the occurrence of traffic accidents. These findings are consistent with sociotechnical approaches that emphasize the relevance of the organizational factors and its influence on safety [4,6,7,8,9]. Moreover, it is also congruent with previous research conducted in other complex systems (e.g., healthcare or construction) that suggest that organizational learning benefits safety [16,17,18,19,20,21,22,23,24]. Our findings extend previous research by means of examining the benefits of organizational learning in the road transport industry.

Hypothesis 2 posited that training would be negatively related to traffic accident occurrence in transport organizations. Results did not support this hypothesis. A potential explanation for the unexpected results could be the need to take into account other factors that might moderate this relationship. For instance, the influence of training on accident occurrence might depend on the quality of the training or their scope (e.g., knowledge, skills, reinforcing attitudes or values) [29]. These questions remain unexplored. Another potential explanation could be the operationalization of the dependent variable. Some authors [28] claim that one of the difficulties in the assessment of training effects on the occurrence of traffic accidents is the low variance of the accident variable that makes difficult to detect if there is indeed an effect of training. In addition, drivers who have been trained might be involved in an accident without being necessarily culpable; others might have caused the accident [28]. Thus, the accident variable should only contain culpable crashes in order to obtain a purer measure of the intended construct and subsequently larger effect sizes. These issues might explain why previous research has failed to support the postulated benefits of training on driving performance [27,28,29,30].

This study has a number of theoretical implications. Findings supported theoretical postulates on the need to take into account organizational factors such as organizational learning to better understand road safety [6,7,8]. In this sense, it encourages future studies to move toward more complex models of traffic accident occurrence in road transport organizations.

Our findings also have important practical implications to prevent road traffic accidents and its fatal consequences. Results suggest that policies and practices on road safety should promote organizational learning. Furthermore, it encourages future analysis of traffic accidents related to road transport and, subsequent interventions, to avoid the adoption of merely driver-focussed countermeasures [4], and to take into account organizational factors such as organizational learning.

With respect to limitations, findings are based on self-reported measures which might lead to common method variance. However, common method variance can be prevented by means of using properly developed instruments [35] as the ones included in this study. In fact, findings showed that organizational learning and training were differentially related to the occurrence of traffic accidents. These results would be unlikely as a consequence of common method variance [36].

Second, a longitudinal design would be more appropriate to establish causal inferences. Finally, it is difficult to detect whether organizational factors have an impact on traffic accidents occurrence due to the low variability of this dependent variable [28]. Future studies should consider yearly accident rate per organization and other dependent variables that present more variability such as near-accidents or traffic offenses. Yearly accident rate per organization is a more reliable measure of traffic accidents in transport organizations. However, organizations are reluctant to provide objective measures of accident rates to others. Many organizations consider this information to be compromising. Alternatively, we have addressed traffic accidents occurrence and we have controlled for the effect of organizational size.

Another avenue for future research is to take into account other dependent variables that reflect success (safe performance) rather than failure (poor performance or traffic accidents). These relationships could also be tested in other transport modes (e.g., air, sea, rail…). Finally, future studies should develop a more comprehensive model that takes into account potential intervening variables in the relationship between organizational factors and road safety. These intervening variables might explain the unexpected non-significant relationships found in the current study between training and the occurrence of accidents. 

## 5. Conclusions

In conclusion, promoting organizational learning in road transport organizations reduces the likelihood of traffic accidents in these organizations. These findings support the postulates of the sociotechnical approach in several ways. First, it points out that not only governments but also the organizations can help to reduce traffic accidents in the road transport of goods and passengers. Second, it encourages future research to go beyond technological and regulatory factors that influence road safety to take into account organizational processes such as organizational learning. These findings have relevant practical implications due to human and socio-economic costs associated with work-related traffic accidents. Future research should further explore the relationship between training and traffic accidents in road transport organizations. In this study this relationship turned to be non-significant.

## Figures and Tables

**Table 1:** Sample characteristics (N = 107 organizations).

Variable	Categories	Frequency	Percentage
Organizational size	Small (less than 50 employees)	88	82.2
	Medium (between 50 and 250)	16	15
	Big (more than 250)	3	2.8
Type of transport	Goods	65	60.7
	Passengers	42	39.3

**Table 2:** Correlations among the variables included in the study.

	1	2	3	4	5
1. Organizational size	-				
2. Type of transport	0.14	-			
3. Organizational learning	0.02	0.01	-		
4. Training	0.08	0.12	0.57 **	-	
5. Occurrence of traffic accidents	0.16	−0.04	−0.21 *	0.002	-

Note. * *p* < 0.05; ** *p* < 0.01; two-tailed tested.

**Table 3:** Organizational factors and traffic accident occurrence. Results of the binary logistic regression model.

Dependent Variable: Traffic Accident Occurrence	Did Any Traffic Accident Take Place in Your Organization Last Year?		OR	95% CI
	**Yes (N = 12)**	**No (N = 95)**		
**Predictors:**				
**Organizational size (OS)**				
OS Small	8 (66.7%)	80 (84.2%)	1.00	
OS Medium	3 (25%)	13 (13.7%)	2.80	0.5–14.8
OS Big	1 (8.3%)	2 (2.1%)	8.60	0.62–118.8
**Type of transport**				
Goods	8 (66.7%)	57 (60%)	1.00	
Passengers	4 (33.3%)	38 (40%)	0.48	0.11–2.06
**Organizational Learning**			0.33	0.14–0.77
**Training**			1.56	0.89–2.75
Constant			0.79	
